# Predicting persistent central serous chorioretinopathy using multiple optical coherence tomographic images by deep learning

**DOI:** 10.1038/s41598-022-13473-x

**Published:** 2022-06-04

**Authors:** Donghyun Jee, Ji Hyun Yoon, Ho Ra, Jin-woo Kwon, Jiwon Baek

**Affiliations:** 1 Department of Ophthalmology, St. Vincent Hospital, College of Medicine, The Catholic University of Korea, Suwon, Gyeonggi-do Republic of Korea; 2 Department of Ophthalmology, Bucheon St. Mary’s Hospital, College of Medicine, The Catholic University of Korea, #327 Sosa-ro, Wonmi-gu, Bucheon, Gyeonggi-do 14647 Republic of Korea; 3 Department of Ophthalmology, College of Medicine, The Catholic University of Korea, Seoul, Republic of Korea

**Keywords:** Medical research, Translational research, Retinal diseases, Eye manifestations

## Abstract

We sought to predict whether central serous chorioretinopathy (CSC) will persist after 6 months using multiple optical coherence tomography (OCT) images by deep convolutional neural network (CNN). This was a multicenter, retrospective, cohort study. Multiple OCT images, including B-scan and *en face* images of retinal thickness (RT), mid-retina, ellipsoid zone (EZ) layer, and choroidal layer, were collected from 832 eyes of 832 CSC patients (593 self-resolving and 239 persistent). Each image set and concatenated set were divided into training (70%), validation (15%), and test (15%) sets. Training and validation were performed using ResNet50 CNN architecture for predicting CSC requiring treatment. Model performance was analyzed using the test set. The accuracy of prediction was 0.8072, 0.9200, 0.6480, and 0.9200 for B-scan, RT, mid-retina, EZ, and choroid modalities, respectively. When image sets with high accuracy were concatenated, the accuracy was 0.9520, 0.8800, and 0.9280 for B-scan + RT, B-scan + EZ, and EZ + RT, respectively. OCT B-scan, RT, and EZ *en face* images demonstrated good performances for predicting the prognosis of CSC using CNN. The performance improved when these sets were concatenated. The results of this study can serve as a reference for choosing an optimal treatment for CSC patients.

## Introduction

Central serous chorioretinopathy (CSC) is a common retinal disease characterized by serous detachment of the neurosensory retina^1^. Most cases of CSC are idiopathic and regress spontaneously within 3 to 4 months^2^. However, the disease is non-resolving and chronic in some patients, and clinicians’ ability to predict the prognosis of the natural course and whether the disease will become chronic and require treatment is limited due to its multifactorial etiology and complex pathogenesis.

Optical coherence tomography (OCT) images provide broad anatomical information on the retinal and choroidal changes that take place in CSC. Numerous previous studies have reported that these characteristic changes differ among CSC types and have suggested the use of these features for prognosis of CSC patients. Some examples of these characteristic changes involve the hyperreflective choroidal vessel wall, the photoreceptor outer segment and ellipsoid zone, and choroidal and macular thickness^3–5^.

In recent years, artificial intelligence using deep learning (DL) has been actively applied to many areas of retinal imaging, including diabetic retinopathy and age-related macular degeneration (AMD)^6–8^. DL has been demonstrated to distinguish acute and chronic CSC when using single or multiple OCT B-scan images^9,10^. The OCT imaging system provides not only B-scans, but also *en face* images, which offer layer-by-layer information on the total macular area. In the current study, we assessed the feasibility of a DL model trained using multiple OCT images for prognosis of CSC and whether the disease will persist after 6 months.

## Results

### Participant demographic and clinical characteristics

In total, 832 eyes from 832 patients were included in the study (593 self-resolving and 239 persistent cases). The mean ages of the self-resolving CSC and persistent CSC groups were 52.00 ± 10.67 years and 54.73 ± 11.93 years, respectively (P = 0.014). The mean baseline BCVA and BCVA at 6 months were better in the self-resolving group (both P < 0.001). The mean CMT was higher in the persistent group (P < 0.001). The sex distribution, laterality, and baseline SFCT did not reveal significant differences between groups (P = 0.920, 0.836, and 0.585, respectively). These clinical features are summarized in Table 1.

**Table 1:** Demographic and clinical information of the study participants.

Features	Self-resolving CSC (n = 593)	Persistent CSC (n = 239)	P-value
Age (years), mean ± SD	52.00 ± 10.67	54.73 ± 11.93	0.014
Sex (male %)	78	79	0.797
Laterality (right eye %)	46	47	0.830
SFCT (µm), mean ± SD	399.35 ± 79.27	404.16 ± 91.4	0.565
CMT (µm), mean ± SD	352.29 ± 113.92	450.26 ± 182.78	< 0.001
Baseline BCVA (logMAR), mean ± SD	0.14 ± 0.2	0.27 ± 0.27	< 0.001
BCVA at 6 months (logMAR), mean ± SD	0.08 ± 0.16	0.14 ± 0.21	< 0.001

CSC: central serous chorioretinopathy; SD: standard deviation; SFCT: subfoveal choroidal thickness; CMT: central macular thickness; BCVA: best-corrected visual acuity; logMAR: logarithm of the minimal angle of resolution.

### Performance of deep learning models for predicting persistent CSC

The accuracy values were 90.36%, 93.60%, 73.60%. 96.00%, and 76.80% for the OCT image validation sets of B-scan, retinal thickness, mid-retinal, EZ, and choroid, respectively.

For the test set, the accuracy percentages were 80.72%, 92.00%, 64.80%, 92.00%, and 63.20% for B-scan, retinal thickness, mid-retinal, EZ, and choroid, respectively. The F1 scores by set were 0.6800, 0.8718, 0.3125, 0.8718, and 0.1154, respectively. The accuracy, precision, recall, specificity, F1 score, and kappa values for each model are summarized in Table 2.

**Table 2:** The performance of the deep learning models trained using each OCT image set for CSC prognosis.

	Accuracy	Precision	Recall	Specificity	F1 score	Kappa mean	Kappa SE
B-scan	0.8072	0.7391	0.6296	0.8929	0.6800	0.782	0.060
Retinal thickness	0.9200	0.9189	0.8293	0.9643	0.8718	0.814	0.056
Mid-retinal	0.6480	0.4348	0.2439	0.8452	0.3125	0.100	0.087
Ellipsoid zone	0.9200	0.9189	0.8293	0.9643	0.8718	0.814	0.056
Choroid	0.6320	0.2727	0.0732	0.9048	0.1154	0.027	0.064

OCT: optical coherence tomography; CSC: central serous chorioretinopathy; SE: standard error.

F1 score = 2 × {(precision × recall)/(precision + recall)}.

Upon concatenation of the two highest performing image sets, the accuracy percentages for the validation set were 96.80%, 86.40%, and 96.00%, for B-scan + EZ, B-scan + retinal thickness, and EZ + retinal thickness, respectively. For the test set, the accuracy was 95.20%, 88.00%, and 92.80% for each set, respectively. The accuracy, precision, recall, specificity, F1 score, and kappa values for each model trained using concatenated images are summarized in Table 3.

**Table 3:** The performance of the deep learning models trained using concatenated OCT image sets for CSC prognosis.

	Accuracy	Precision	Recall	Specificity	F1 score	Kappa mean	Kappa SE
B-scan + retinal thickness	0.9520	0.9487	0.9024	0.9762	0.9250	0.7230	0.0670
B-scan + EZ	0.8800	0.8421	0.7805	0.9286	0.8101	0.8900	0.0440
EZ + retinal thickness	0.9280	0.881	0.9024	0.9405	0.8916	0.8380	0.0520

OCT: optical coherence tomography; CSC: central serous chorioretinopathy; SE: standard error; EZ: ellipsoid zone.

F1 score = 2 × {(precision × recall)/(precision + recall)}.

**Figure 1:**
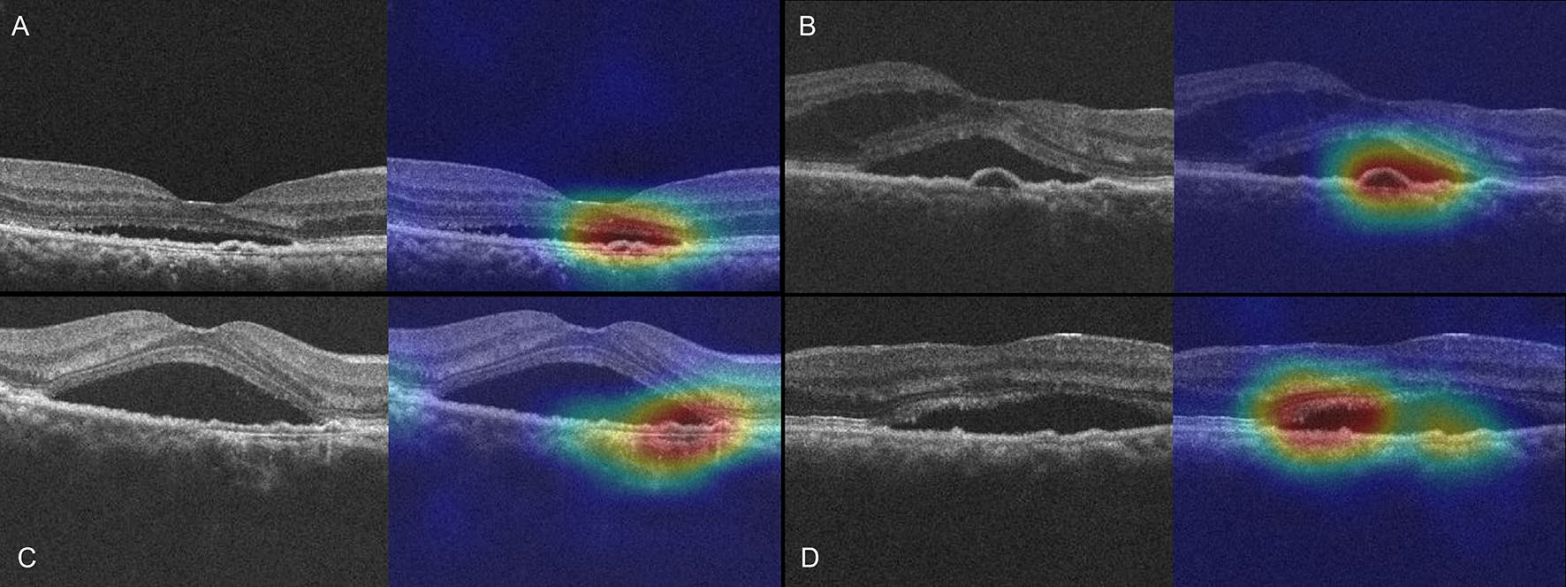
Four representative cases of persistent Grad-CAM analysis on B-scan. (**A**–**D**). The heatmaps highlight areas of shallow irregular pigment epithelial detachment in all cases. Areas of increased reflectivity representing elongated photoreceptor outer segments in the subretinal fluid area are shown in (**D**).

**Figure 2:**
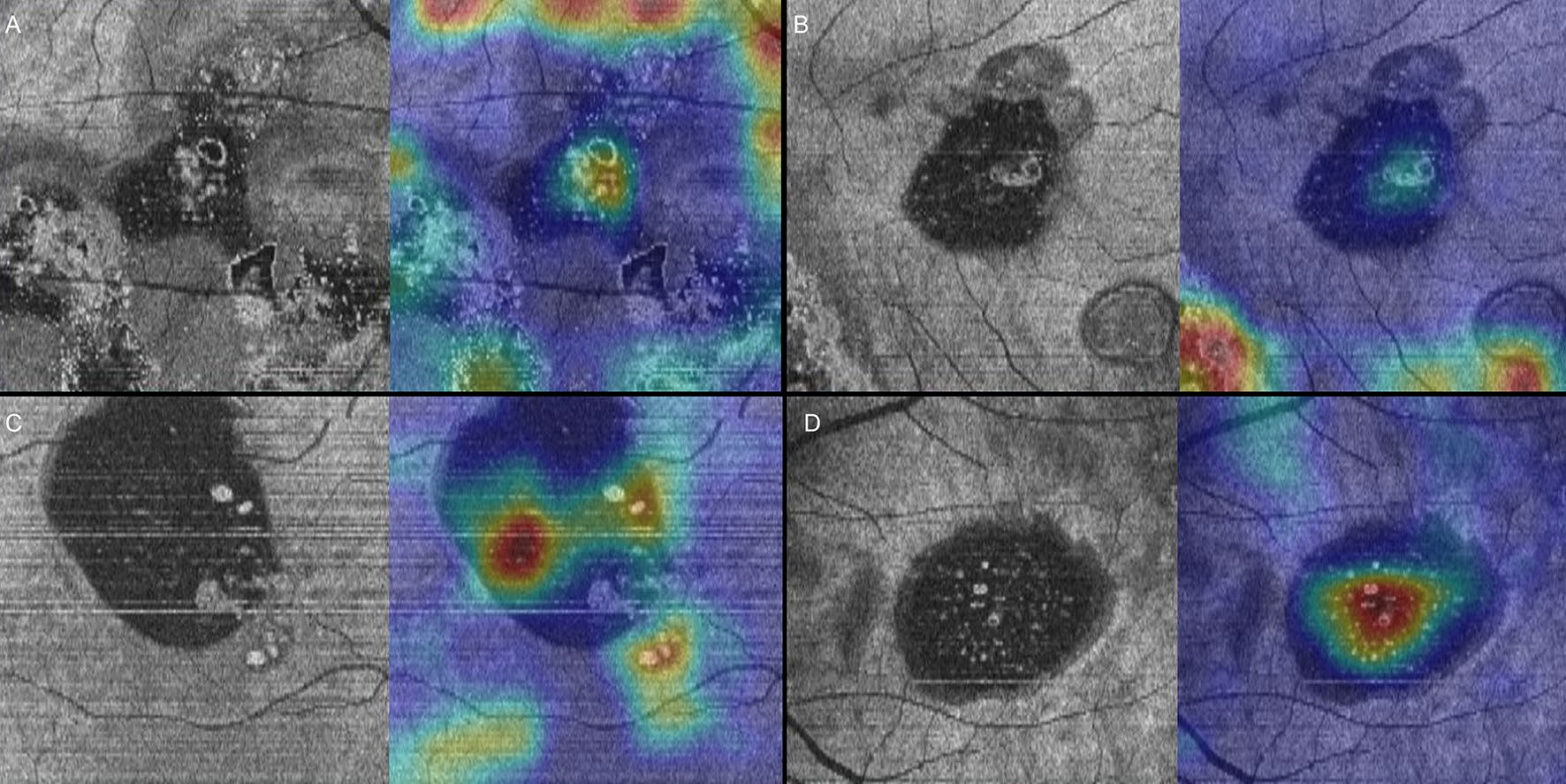
Four representative cases of persistent Grad-CAM analysis on OCT *en face* images of the ellipsoid zone (EZ) layer. (**A**–**D**) A heatmap overlying the OCT *en face* image of the EZ layer highlights areas of the neurosensory retinal detachment with or without outer retinal defects within the main lesion in all cases. Areas of the neurosensory retinal detachment with or without outer retinal defects outside the main lesion are important in Cases A and B.

**Figure 3:**
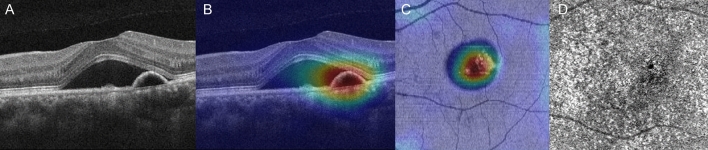
A representative case of persistent CSC with OCT angiography: the left eye of a 45-year-old male. (**A**) The OCT B-scan shows SRF with shallow irregular PED. (**B**) The heatmap overlying the OCT B-scan highlights important areas for use of DL in determining the prognosis of this case as persistent CSC. (**C**) A heatmap overlying the OCT *en face* image of the ellipsoid zone layer highlights the important areas. (**D**) OCT angiography does not reveal any choroidal neovascularization.

Heatmap analysis with Grad-CAM highlighted areas of shallow irregular pigment epithelial detachment (PED) and areas of increased reflectivity beneath the detached retina as important area (Fig. 1) on OCT B-scan. Also, areas of the neurosensory retinal detachment with or without outer retinal defects are highlighted on OCT *en face* images of EZ layer (Fig. 2). Optical coherence tomography angiography was available in some cases of persistent CSC. In these cases, areas of PED containing RPE undulation (retinal pigment epithelium) on B-scan was revealed to be an important area for the decision but did not conceal choroidal neovascularization (Fig. 3).

## Discussion

In this study, we developed DL models to predict the prognosis of CSC patients and whether the disease will be self-resolving or persistent for the next 6 months. The results of our study showed that DL model training using pretrained ResNet50 can predict the prognosis of CSC eyes by analyzing OCT B-scans and *en face* images of retinal thickness, EZ layer, and choroid layer with good performance. In addition, the performance of DL models was better when these image sets were concatenated.

Diagnosis, classification, and prognosis prediction of a disease using DL has become an active field of imaging research in ophthalmology, especially the retina^6,8–13^. In CSC, diagnosis and classification of the disease, detection of subretinal fluid, and prediction of post-therapeutic visual acuity have been investigated using DL^9,10,12–15^. Most previous studies have used fundus photography or OCT B-scans alone for development of DL models. The value of *en face* images in CSC has been emphasized by numerous studies^12,16–18^. The pathologic features of the RPE and choroid in CSC eyes are well visualized on *en face* images^19^. Therefore, this study utilized multi-layer OCT *en face* images along with B-scan images for automated prediction of the prognosis of CSC.

In predicting the prognosis of CSC patients, OCT image sets of B-scans, retinal thickness, and the EZ layer showed good performance in this study. Xu et al*.* applied DL to predict visual acuity in CSC patients using clinical features, collected features from OCT B-scan images, and showed that the DL model could predict the visual outcome of CSC after treatment with low mean absolute error^13^. This study confirmed that OCT B-scan images can be used to predict the outcome of CSC using a DL model. Nonetheless, the recall and F1 scores produced by the DL model using B-scans alone were unsatisfactory.

*En face* image sets of the EZ and retinal thickness showed better accuracy, sensitivity, and specificity in DL prediction models of CSC prognosis. The *en face* EZ layer reflects changes in the outer or inner photoreceptor segment as well as the EZ (IS/OS junction). Significant loss in photoreceptors and an elongated photoreceptor outer segment length are characteristic findings and are also associated with visual prognosis in patients with chronic CSC^4,20,21^. The good performance of the DL model using the EZ layer *en face* images suggests that changes to the adjacent EZ layers play an important role in the prognosis of CSC.

The integrity of EZ observed on OCT B-scan and CMT was shown to be important in predicting visual acuity at 1, 3, and 6 months^13^. OCT volume scans were used to estimate post-treatment retinal function in CSC patients in a study by Pfau et al*.*^15^. Their study showed that localized retinal sensitivity after treatment can be inferred from the thicknesses of the retinal layers in CSC eyes using machine learning (random forest after feature extraction). Similarly, in this study, the thickness map of the entire retinal layer, which contains information on the thicknesses of retinal layers and CMT, could predict the prognosis of CSC using DL. It is not clear whether higher CMT or shallow diffuse CMT was prognostic factor for persistent CSC in this study. Arora et al. showed that shallow subretinal fluid was associated with recurrent or complicated CSC^22^. This might be the point for predictionin this model, but this interpretation requires caution since the association between CMT and visual outcome depends on types of CSC. An *en face* image of the mid-retinal layer did not show good results for training the DL model.

For *en face* OCT images of the choroidal vasculature, CSC shows characteristic changes of diffuse distribution of dilated Haller vessels^17,23,24^. Aoyama et al*.* demonstrated that CSC can be diagnosed by DL with OCT *en face* slab showing the choroidal vasculature^12^. Nonetheless, *en face* images of the choroid were not useful for a DL model to predict the outcome of CSC. Although a previous report implied different characteristics of the choroidal vasculature between acute and chronic CSC, we suggest that these features are not significant enough for use in a DL prediction model^16^.

A Grad-CAM heatmap analysis of OCT B-scan images showed that the hyperreflective area beneath the detached retina was important for determining the prognosis of the eye as persistent. The hyperreflective area indicates an elongated photoreceptor outer segment, which is well recognized as an important feature of chronic CSC^25^. In addition, shallow irregular PED areas were highlighted on the heatmaps. A small, flat, irregular PED could be a marker of chronic or recurrent CSC and implies genetic overlap of patients with AMD, which can explain the refractoriness of the condition^26,27^.

Important limitations of this study include the limited performance according to the OCT device as this study only included images obtained with one HD-OCT system. Currently, high performance of a DL model requires input data from the same OCT system. Additionally, although we used a recent consensus for CSC classification that was developed using multimodal imaging^28^, there might be some controversy in defining CSC as acute or persistent in the CSC eyes that did not resolve during months three to five. However, we believe that there will be no controversy in that the disease is considered refractory if it persists for more than 6 months; predicting this entity at baseline can be useful for both doctors and patients. Further research involving multimodal images from many OCT machines and more retinal specialists are warranted to validate the results of the current investigation.

This study was unique in that it assessed the feasibility of a DL model using multiple OCT imaging sets for predicting the prognosis of CSC, which few previous studies have done. OCT volume scan contains a full set of B-scan images and *en face* image can be obtained from the volume scan. Using the full set of B-scam images may provide more information on the status of retina and choroid. However, the volume scan contains up to hundreds of B-scan images, which requires tremendous resources and time for training and validating using CNN. The DL models developed in this study utilized OCT slabs that were automatically provided by the commercial OCT viewer program and can easily be applied in practice. And *en fac*e images used in the study contains information which had been proved to be valuable in CSC. Further progression in computational limit may enable using whole set of images and improve the performance of DL models.

In conclusion, this study assessed the performance of DL models for CSC prognosis using multiple OCT image sets. The results revealed good performance of DL models created using OCT B-scans, retinal thickness, and EZ *en face* images. Concatenation of these image sets increased the performance of a DL model. Heatmaps revealed that a shallow irregular PED and an elongated photoreceptor outer segment on B-scans and a defective *en face* EZ area were important areas for prognosis. This automated prediction system will aid in individualized medical care for patients with CSC.

## Methods

This study was approved by the Institutional Review Board of Bucheon St. Mary’s Hospital, which waived the need for written informed consent because of the study’s retrospective design. The study was conducted in accordance with the tenets of the Declaration of Helsinki.

### Participants

The inclusion criteria were consecutive patients 18 years or older who visited Bucheon St. Mary’s or St. Vincent Hospital between April 2016 and January 2022 and were diagnosed with CSC. Diagnosis was determined using high-definition OCT (Cirrus 4000 or Cirrus 6000; Carl Zeiss Meditec, Jena, Germany) and fluorescein angiography. OCT angiography image was assessed for presence of neovascularization in available patients. In patients with bilateral disease, the eye with the most severe symptoms was chosen for the study. Clinical information of age, sex, laterality of the diseased eye, baseline best-corrected visual acuity (BCVA), and BCVA at 6 months was collected from a chart review.

The exclusion criteria were (1) concurrent macular disease or scarring other than CSC (e.g., AMD, punctate inner choroidopathy, vitelliform dystrophy); (2) history of previous treatment (e.g., photodynamic therapy, laser photocoagulation, intraocular injections, periocular injections, and systemic treatment); (3) high myopia (> − 6.00 diopters or axial length > 26 mm); and (4) poor image quality (image quality < 7 or significant artifacts).

### Image preparation, model training, and performance metrics

All patients underwent HD-OCT line scanning and volume scanning at baseline. These images were labeled as self-resolving or persistent based on the recent consensus on CSC classification by a CSC international group (i.e., patients presented with subretinal fluid more than 6 months after baseline)^28^. The presence of subretinal fluid was evaluated using the full set of OCT B-scan at the macular area at 6 months from the baseline. The horizontal HD line OCT B-scan, which crossed the fovea, and each *en face* image of retinal thickness, mid-retinal, ellipsoid zone (EZ), and choroidal layers were saved as a JPEG (.jpg) file for each patient. Central macular thickness (CMT) was obtained from the retinal thickness map of Cirrus HD-OCT review software (version 10, Carl Zeiss Meditec), and subfoveal choroidal thickness was manually measured as the vertical distance between Bruch’s membrane and the chorio-scleral border under the fovea on an OCT line scan.

Images underwent resizing and preprocessing. The B-scan image was cropped to exclude irrelevant areas, resized to 261 × 351 × 3 pixels, and underwent contrast adjustment. All *en face* images were cropped to remove the border, resized to 396 × 396 × 3 pixels, and underwent contrast adjustment. Each image set was randomly divided into 70% training, 15% validation, and 15% test sets.

**Figure 4:**
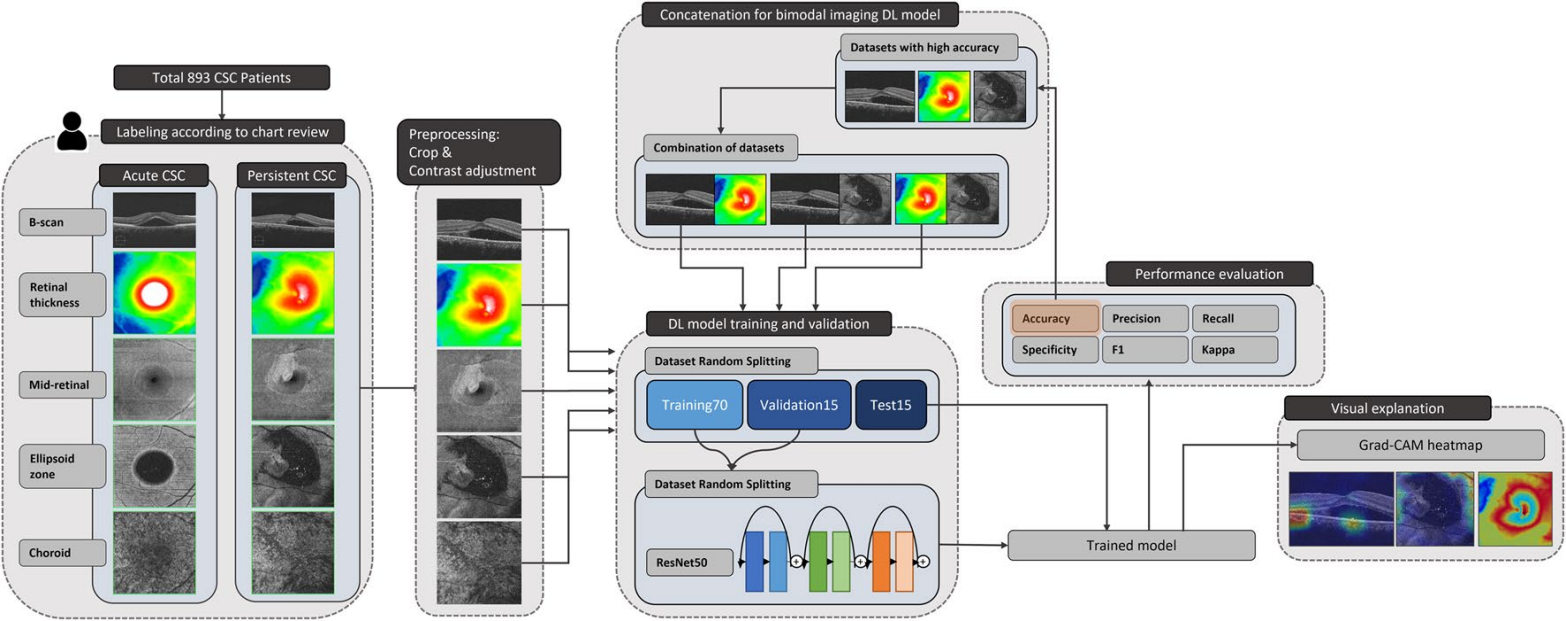
A schematic of deep learning (DL) model training, validation, and performance assessment for prognosis of central serous chorioretinopathy (CSC) using optical coherence tomography (OCT) image sets. Baseline horizontal OCT B-scan crossing the fovea and *en face* images of retinal thickness, mid-retinal, ellipsoid zone (EZ), and choroidal layers were saved as a JPEG (.jpg) file from each patient and labeled as acute or persistent disease status at 6 months from baseline. The collected images underwent resizing and preprocessing of crop and contrast adjustment. Then, a deep learning model using ResNet50 architecture was trained and validated to predict the impact on the training and validation sets. Next, a performance evaluation on the test set was carried out. Visual explanation analysis was performed using a heatmap generated with gradient-weighted class activation mapping (Grad-CAM). Image sets with high accuracy were concatenated for bimodal imaging model training.

The models were trained using pretrained ResNet50 architecture on MATLAB 2021b (MathWorks, Inc., Natick, MA, USA) for each image set (B-scan, retinal thickness, mid-retinal, EZ, and choroid). Selection of deep and shallow convolutional neural network (CNN) architectures was based on our previous studies on DL of OCT images for macular diseases^29,30^. To evaluate the performance of binodal imaging, two OCT images from each set underwent concatenation, and the resulting image was used for model training, validation, and testing. All experiments were conducted on a desktop computer equipped with NVIDIA RTX 3090 and Intel i9 CPUs. Each model was trained for 25 epochs with a minibatch size of 32 and an initial learning rate of 0.01. The performance of each model was assessed using validation accuracy and accuracy, precision, recall (sensitivity), specificity, F1 score, and kappa score on the test set. To determine the focus of the DL model in the image, we analyzed the heatmap generated by gradient-weighted class activation mapping (Grad-CAM)^31^. Grad-CAM heatmaps were generated using ‘gradCAM’ function implemented in MATLAB. Image preprocessing, model training, and performance evaluation processes are schematized in Fig. 4.

### Statistics

Statistical analysis was performed using MATLAB 2021b. T-tests were used to compare clinical features between groups, and the chi-square test was used to compare categorical variables. Accuracy, precision, recall, specificity, and F1 scores were calculated for each model. The kappa score mean and standard deviation (SD) were used to determine the agreement between truth and each model. Continuous variables are presented as mean ± SD.

### Ethics approval and consent to participate/publication

This study was conducted in accordance with the tenets of the Declaration of Helsinki. The study was approved by the Institutional Review Board of Bucheon St. Mary’s Hospital, which waived the need for written informed consent because of the study’s retrospective design (HC20TISI0091).

## Data Availability

The datasets generated and/or analyzed during the current study are available from the corresponding author upon reasonable request.
